# Effects of Noncontact Shoulder Tool Velocities on Friction Stir Joining of Polyamide 6 (PA6)

**DOI:** 10.3390/ma15124214

**Published:** 2022-06-14

**Authors:** Raheem Al-Sabur, Hassanein I. Khalaf, Aleksandra Świerczyńska, Grzegorz Rogalski, Hesamoddin Aghajani Derazkola

**Affiliations:** 1 Mechanical Department, Engineering College, University of Basrah, Basrah 6100, Iraq; hassanein.khalaf@uobasrah.edu.iq; 2 Faculty of Mechanical Engineering and Ship Technology, Institute of Manufacturing and Materials Technology, Gdańsk University of Technology, Gabriela Narutowicza Street 11/12, 80-233 Gdańsk, Poland; aleksandra.swierczynska@pg.edu.pl (A.Ś.); grzegorz.rogalski@pg.edu.pl (G.R.); 3 Department of Mechanical Engineering, Islamic Azad University of Nour Branch, Nour 21655432, Iran

**Keywords:** friction stir welding, noncontact shoulder, simulation, polyamide 6

## Abstract

In this study, the effects of the traverse and rotational velocities of the noncontact shoulder tool on the heat generation and heated flux during the friction stir joining of high-density polyamide 6 (PA6) polymer were investigated. The computational fluid dynamics (CFD) method was employed to simulate the thermomechanical phenomena during the friction stir joining (FSJ) process of PA6. A developed model was used to consider the void formation and thermochemical properties of PA6. The surface and internal heat flow, material flow, and geometry of the joint were simulated, and an experimental study evaluated the simulation results. The simulation results indicated that the stir zone formed was smaller than regular joints with a noncontact shoulder tool. Despite the polymer’s traditional FSJ, heat generation and material flow do not differ significantly between advancing and retreating sides. On the other hand, the surface flow is not formed, and the surface temperature gradient is in a narrow line behind the tool. The material velocity increased at higher rotational speed and lower transverse velocity and in the stir zone with more giant geometry forms. The maximum generated heat was 204 °C, and the maximum material velocity was predicted at 0.44 m/s in the stir zone, achieved at 440 rpm and 40 mm/min tool velocities.

## 1. Introduction

Friction stir joining (FSJ) is an advanced joining process compared to other welding methods with many advantages, including low cost, high flexibility, no need for a skilled operator, and no contamination [1,2]. It is commonly used to join various metals in the production of similar and dissimilar joints [3]. Generally, the FSJ tool consists of a shoulder and a pin [4,5]. In this method, a rotational non-consumable tool penetrates the joint line and generates heat with frictional contact with the base materials [6,7,8]. This heat, called frictional heat, converts the base materials to plasticized form and mixes them. In this process, the contact area of the tool and base materials is critical because heat generation is related to the frictional contact area [9,10]. It is shown that the primary frictional heat is generated by the FSJ tool shoulder [11,12].

Polyamides belong to polymers that are thermally stable and wear-resistant. Furthermore, they have good shaping properties and low density. PA 6, trade-named as nylon, is a semicrystalline polyamide widely used by many industries, inter alia the automotive sector [13,14].

Recently, the FSJ method was used to join nonmetallic materials, allowing this method to cover a broader range of materials. In this regard, researchers have shown that, compared with other joining processes, FSJ can improve mechanical properties [15,16]. The variety of polymers that can be subjected to FSJ is not comprehensive. Mendes et al. [17] investigated and optimized the feasibility of acrylonitrile butadiene styrene (ABS) joining with FSJ. Simões et al. [18] implemented the FSJ process to join polymethyl methacrylate (PMMA). They show that the formed joint regions at the FSJ of PMMA were different from the FSJ of metallic materials. In another study, Azarsa and Mostafapour [19] used the FSJ technique to join high-density polyethylene (HDPE). They concluded that the joining defects decrease at the tool’s high rotational speed and low traveling velocity, and the final joint strength increases. Saeedy and Givi [20] examined the influence of process parameters on the quality of HDPE joints. They stated that, after optimization of FSJ parameters, the produced joint reached 75% strength of HDPE.

Panneerselvam and Lenin [21] examined the feasibility of joining PA6 by the FSJ process and showed that the joining process is possible. In another research, Panneerselvam and Lenin [22] showed that the pin thread and rotational direction of the FSJ tool could significantly affect the final product quality. These effects were also reported in the FSJ of polypropylene (PP) sheets. It was shown that the threaded pin can reduce cavities and inclusions in the joint line of PP.

Due to the sensitivity of polymer structures, there are various factors in the FSJ of these materials that are not understood for optimum joint properties [23]. For this reason, researchers tried to use simulation methods to find the polymer’s behavior during the FSJ process. Because of the intense plastic deformation and high strain rate of polymers in this process, simulating these types of materials during FSJ proved extremely difficult. The available literature on this topic is very limited [24,25].

Aghajani Derazkola et al. simulated FSJ of PMMA and polycarbonate (PC) using a computational fluid dynamics (CFD) model [2,26,27]. They used CFD to find the optimum heat generation that maximizes tensile strength during the FSJ process. Sheikh-Ahmad et al. [28,29] simulated a similar FSJ of HDPE. Shete and Yarasu [30] used the finite element method (FEM) to simulate a high-density polyethylene joint’s friction stir spot welding (FSSW). According to the available literature, various polymers and FSJ processes have not been thoroughly analyzed. Simulation of the FSJ process helps to understand the thermomechanical phenomena that physical techniques cannot measure. For this reason, this article aimed to investigate thermomechanical phenomena during the noncontact shoulder FSJ of the PA6.

## 2. Process Modelling

### 2.1. Model Description

In this study, a domain was defined for the simulation of the process. The simulation domain consisted of the welding material and various FSJ tools. The analysis of material flow was in three dimensions (3D). During the simulation process, the forward motion of the FSJ tool was considered preliminarily, and tool exit steps were ignored in this study. Due to the assumptions, a steady-state coupled material flow was employed to simulate this process. The dimensions of FSJ tools and the size of the workpiece were selected according to the actual size. In the simulation domain, the FSJ tool moving direction was set to the *x*-axis, and the *z*-axis was set as the normal axis. The 3D material flow was employed to increase the accuracy of simulation results. This study defines the material velocity in the *x*, *y*, and *z* directions by the *g*, *h*, and *f* signs. The simulation domain was modeled in ANSYS FLUENT software, and equations were solved by the CFD package. The PA6 workpiece was selected as a non-Newtonian single-phase fluid, and conservation equations for continuity, momentum, and energy were used to simulate the material flow. The continuity and energy equations are presented in Equations (1) and (2), respectively [31].

(1) $$\frac{dg}{dx}+\frac{dh}{dy}+\frac{df}{dz}=0.$$

(2) $$\rho c\left(\frac{\partial T}{\partial t}+g\frac{\partial T}{\partial x}+h\frac{\partial T}{\partial y}+f\frac{\partial T}{\partial z}\right)+\dot{H}=k\left(\frac{\partial^{2}T}{\partial x^{2}}+\frac{\partial^{2}T}{\partial y^{2}}+\frac{\partial^{2}T}{\partial z^{2}}\right).$$

The momentum equations in the *x*-, *y*-, and *z*-directions are presented below [32,33].

(3) $$\frac{\partial g}{\partial t}+g\frac{\partial g}{\partial x}+h\frac{\partial g}{\partial y}+f\frac{\partial g}{\partial z}=F_{x}-\frac{1}{\rho }\frac{\partial P}{\partial x}+\nu \left(\frac{\partial^{2}g}{\partial x^{2}}+\frac{\partial^{2}g}{\partial y^{2}}+\frac{\partial^{2}g}{\partial z^{2}}\right).$$

(4) $$\frac{\partial h}{\partial t}+g\frac{\partial h}{\partial x}+h\frac{\partial h}{\partial y}+f\frac{\partial h}{\partial z}=F_{y}-\frac{1}{\rho }\frac{\partial P}{\partial y}+\nu \left(\frac{\partial^{2}h}{\partial x^{2}}+\frac{\partial^{2}h}{\partial y^{2}}+\frac{\partial^{2}h}{\partial z^{2}}\right).$$

(5) $$\frac{\partial f}{\partial t}+g\frac{\partial f}{\partial x}+h\frac{\partial f}{\partial y}+f\frac{\partial f}{\partial z}=F_{z}-\frac{1}{\rho }\frac{\partial P}{\partial z}+\nu \left(\frac{\partial^{2}f}{\partial x^{2}}+\frac{\partial^{2}f}{\partial y^{2}}+\frac{\partial^{2}f}{\partial z^{2}}\right).$$

**Figure 1:**
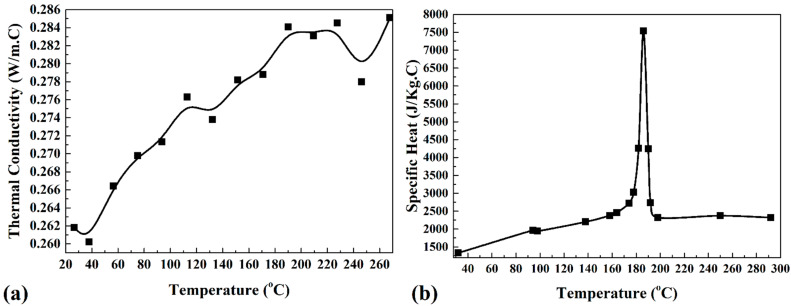
Temperature dependency of (**a**) thermal conductivity and (**b**) specific heat of PA6 [34,35,36,37].

*P*, *ρ*, *T*, *c*, and *k*, represent the local pressure, PA6 density, temperature, specific heat, thermal conductivity, and PA6 viscosity, respectively, in Equations (1)–(5). Figure 1a,b show the specific heat and thermal conductivity of the PA6 used in this study.

### 2.2. Weld Metal Model

**Figure 2:**
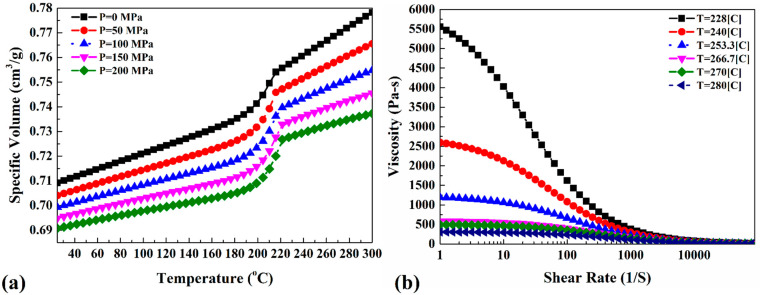
Temperature dependency of (**a**) specific volume and (**b**) viscosity–shear rate of PA6 [34,35,36,37].

Due to the different structures of PA6 and metals, the governing equations of base material properties in FSJ of polymeric materials are different compared to those of metallic ones. In this case, the density of the used PA6 was defined by a specific volume ($\frac{c}{v}$). The specific volume is a property that depends on applied pressure and temperature. This property can be presented by the pressure–volume–temperature (P–V–T) diagram of PA6. This behavior is used because the pressure and temperature in the stir zone are local. The heat transfer coefficient of polymeric materials is low; hence, the pressure and temperature in the stir zone are considered locally. The P–V–T diagram used for PA6 in this study is presented in Figure 2a. The PA6 flow rate was defined as a function of the load, temperature, and time-to-flow function. The polymers should be warmed up until the softening temperature despite the use of metallic materials. At that temperature, they can be stirred using the tools. In this situation, the time to start the softening is presented as “time-to-flow”. The flow rate as a function of load and temperature is presented in Figure 2b.

### 2.3. Boundary Conditions

During simulation of the FSJ process, the total heat ($\dot{H}$ in Equation (2)) is generated by frictional sliding contact (*H_h_*) at the FSJ tool and PA6 interface and plastic deformation (*H_h_*) of PA6 [32,38,39].

(6) $$\dot{H}=H_{h}+H_{p}.$$

The generated heat by sliding friction at the interface of PA6 and the FSJ tool is presented below [40,41].

(7) $$H_{h}=\left[\left(1-\delta \right)\chi \tau +\delta \mu_{f}F_{z}\right]\left(\omega r-g\sin\theta \right).$$

In Equation (7), *r* is the radial distance from the tool axis, and *θ* is the angle with the negative *x*-axis in the counterclockwise direction. *τ* is present as the shear stress of PA6. *δ* is the fractional slip between the FSJ tool and the PA6, *χ* is the mechanical efficiency, *µ_f_* is the friction coefficient, *ω* is the rotational velocity, and *F_z_* is the axial force. The heat generation by plastic deformation (*H_P_*) is presented below [32].

(8) $$H_{p}=\psi \left(\frac{\partial P}{\partial x}+\frac{\partial P}{\partial y}+\frac{\partial P}{\partial z}\right)\left[\begin{array}{l} 2\left({\left(\frac{\partial g}{\partial x}\right)}^{2}+{\left(\frac{\partial h}{\partial y}\right)}^{2}+{\left(\frac{\partial f}{\partial z}\right)}^{2}\right)\\ +{\left(\frac{\partial g}{\partial y}+\frac{\partial h}{\partial x}\right)}^{2}+{\left(\frac{\partial g}{\partial z}+\frac{\partial f}{\partial x}\right)}^{2}+{\left(\frac{\partial f}{\partial y}+\frac{\partial h}{\partial z}\right)}^{2} \end{array}\right].$$

In Equation (8), *ψ* is a constant that specifies the internal mixing of PA6 in the stir zone.

### 2.4. Heat Transfer Model

Due to different contact conditions between the fixture and air (environment) with PA6, various heat transfer models were selected, i.e., the heat transfer equation between PA6 and the backing plate and the heat transfer equation between the surface of the joint line and the environment. The bottom of the PA6 was in direct contact with the backing plate (fixture). As a result, conductive heat transfer of PA6 with the fixture at the bottom surface was taken into account [42].

(9) $$k{\frac{\partial T}{\partial Z}|}_{Bottom}=h_{b}\left(T-T_{a}\right).$$

At the bottom surface, the local temperature governed the heat transfer coefficient according to the following equation [43]:

(10) $$h_{b}=h_{b0}{\left(T-T_{a}\right)}^{0.25}.$$

The joint line and the top surface of the PA6 were in direct contact with air. For this reason, convective and radiation heat transfer models were assumed for those areas.

(11) $$-k{\frac{\partial T}{\partial Z}|}_{Top}=Β\epsilon \left(T^{4}-T_{a}^{4}\right)+h_{t}\left(T-T_{a}\right).$$

An M10 right-hand screw with a length of 4.8 mm without shoulder contact was designed to study the noncontact shoulder FSJ process of PA6. For meshing the domain in this simulation, tetrahedral/hybrid elements with a T-grid shape were chosen. The governing equations were solved using ANSYS FLUENT software (ANSYS, Inc., Canonsburg, PA, USA). The simulation was validated against the experimental results.

**Figure 3:**
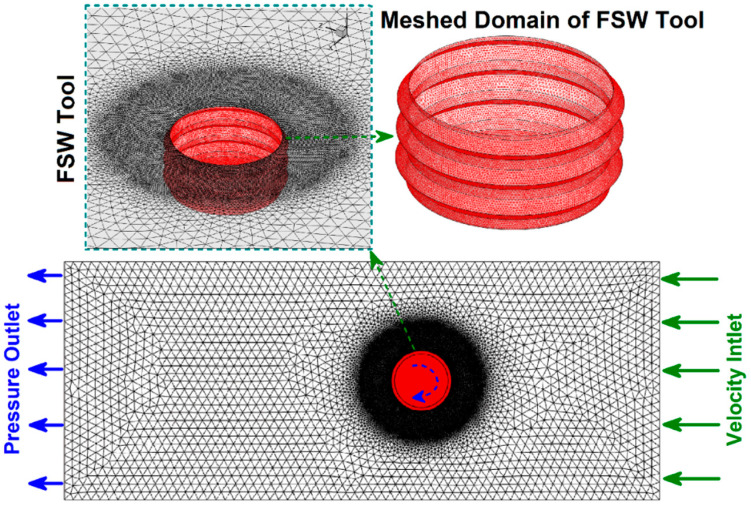
Meshed domain.

Due to the 0.1 mm distance between the tool shoulder and top of the raw sheets, the exiting materials from the joint line were limited. For this reason, the possibility of any upward flow and material exit from the joint line was neglected. Consequently, the overall errors of the simulation (compared to experimental results) were lower than 6%. Figure 3 presents the meshed domain and pin profile used in this study.

## 3. Model Validation

In order to evaluate the results obtained from the simulation, the simulation was validated by experimental tests. In this regard, the obtained data from Inaniwa et al. [44] were used to assess simulation results. They selected polyamide 6 to examine the effect of the rotational tool speed and traveling velocity on the final properties of joints. The contact area between the tool and PA6 was the same as the assumption in the simulation part. The physical and thermal properties of PA6 used by Inaniwa et al. [44] are presented in Table 1.

**Figure 4:**
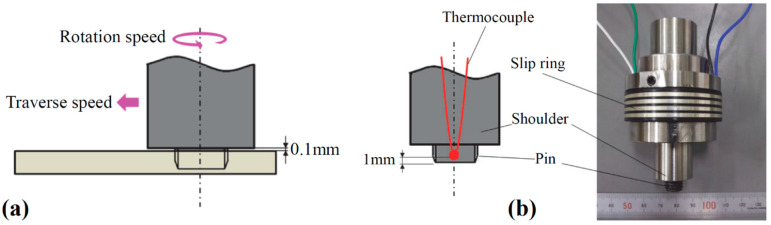
(**a**) Schematic view of noncontact shoulder FSW; (**b**) used FSW tool with thermocouple position [44].

The dimensions of the raw sheet were 100 × 40 × 5 mm with a butt joint configuration. The FSJ shoulder diameter was 20 mm, while an M10 right-hand screw of 4.8 mm length was used as the FSJ pin. The tool had a counterclockwise rotation direction without a tilt angle. During the joining process, the FSJ tool shoulder had a 0.1 mm gap with the surface of the PA6. The FSJ tool had a rotational velocity range between 380 rpm and 500 rpm and a traverse velocity range between 40 mm/min and 50 mm/min. The tool velocity range is presented in Table 2. The k-type thermocouple was located at 1.0 mm from the bottom of the FSJ pin to monitor thermal history during the welding process. A schematic view of the tool and the thermocouple positioning are depicted in Figure 4a,b, respectively.

## 4. Results and Discussion

### 4.1. Heat Generation

**Figure 5:**
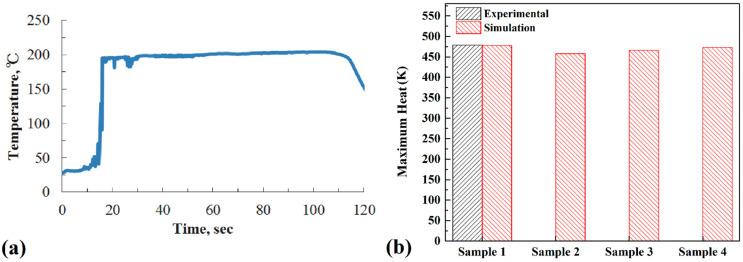
(**a**) Experimental results of sample 1 [44]; (**b**) comparison between maximum heat generation from simulation results and experimental data.

Heat generation during the FSJ process depends on many mechanical factors [45]. The tool angular and traveling velocities are two main factors that can change the total amount of heat generated during the FSJ process [46]. Data from Lnaniwa et al. [44] were used for evaluating the simulation results. The maximum recorded temperature of sample 1 is presented in Figure 5a.

In this study, the maximum heat generated by the tool from simulation results and at different velocities is depicted in Figure 5b. The simulation results revealed that the maximum temperature at samples 1, 2, 3, and 4 was 204 °C, 185 °C, 193 °C, and 200 °C, respectively. In comparison, the maximum recorded temperature was 206.1 °C in experimental sample 1. The comparison of the results indicated a good agreement between the recorded temperature and the simulation output. The difference between the actual result and the simulation output for the maximum temperature in sample 1 was 2 °C. Similar to the heat generation during FSJ, in this study, the results revealed that the amount of heat generation increased with increasing rotational speed and decreased with increasing FSJ tool traverse velocity. Due to the obtained results, the maximum heat generation from 380 rpm to 500 rpm was increased by 15 °C.

**Figure 6:**
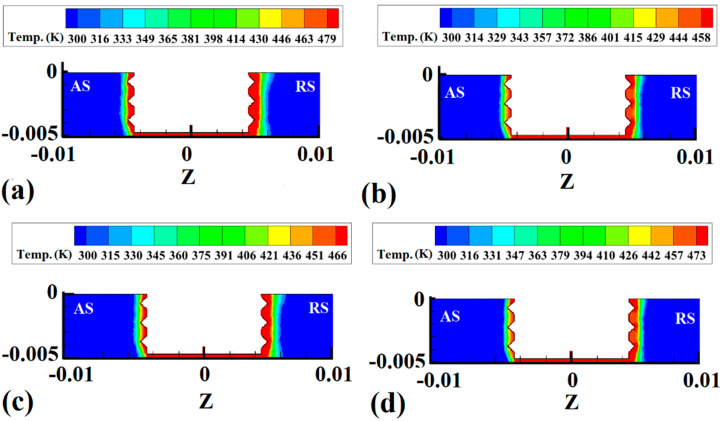
Cross-section view of internal temperature gradient obtained from the simulation results: (**a**) sample 1, (**b**) sample 2, (**c**) sample 3, and (**d**) sample 4.

Simulation results from the internal temperature gradient of samples 1, 2, 3, and 4 are depicted in Figure 6a–d, respectively. The simulation results depict a cross-section view of the joint line. The maximum heat and internal temperature gradient are shown in Figure 6. In regular FSJ of polymers, the tool shoulder directly contacts the raw materials [47]. The shoulder has more of a contact area with the polymer; for this reason, during rotational movement of the FSJ tool, the stirring action in the upper area of SZ is more intense than in other areas. This phenomenon caused the formation of the pelvic shape SZ. In this specific FSJ process, the tool shoulder does not have any contact with PA6. For this reason, the temperature gradient internally and externally is different compared to regular FSJ samples.

The simulation result for the internal temperature gradient indicated that, due to the noncontact shoulder and PA6, the heat diffused around the pin and the temperature gradient was columnar. For this reason, the temperature gradient in the noncontact case was different from the regular FSJ joint of polymeric materials. The tool shoulder did not play any role in heat generation or material flow during this process.

In such a case, the material was stirred by the tool’s pin, and the temperature gradient was limited around the pin. Due to the used threaded pin, the heat generation at the crest of the thread was more intense than in other areas. Due to the low heat friction coefficient of PA6, the internal temperature gradient was not too high. Alternatively, the results revealed that the temperature gradient on the advancing side (AS) was much smaller than that on the retreating side (RS). This phenomenon was due to the rotational direction of the tool. The clockwise direction of the tool caused the PA6 to stretch from AS to RS. In this case, the compressed PA6 in RS concentrated the hot material. There was more hot material in RS compared to the AS case temperature gradient inequality. With increasing tool rotational velocity, the generated heat increased. The results revealed that the heat diffused area increased at higher heat generation.

**Figure 7:**
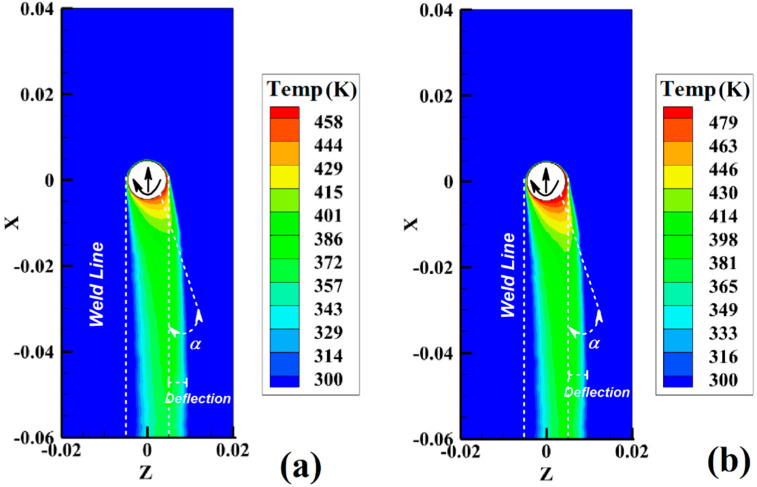
Simulation results from surface temperature gradient of (**a**) sample 1 and (**b**) sample 2 during FSJ of PA6.

Figure 7a,b show the simulation results of surface temperature gradient in sample 1 and sample 2, respectively. During the simulation, these samples were used to study the surface temperature gradient in the cases of maximum heat generation (sample 1) and minimum heat generation (sample 2).

The simulation results showed that the temperature gradient on the surface of PA6 was not comprehensive. As mentioned before, due to the low heat coefficient of PA6, the surface temperature gradient was not similar to the FSJ of metallic materials. The hot region on the surface was narrow. The maximum heat was concentrated in the back of the pin and had a deflection in the RS. With increasing tool rotational velocity and decreasing tool traveling velocity, the total heat generation in SZ increased. Higher heat generation increased the temperature gradient on the surface of the tool. The role of the contact area between the tool shoulder and the surface of the polymer during FSJ is critical. The results indicated that the surface temperature gradient of these samples was different from the normal FSJ process. At the noncontact shoulder joint, the temperature gradient deflected from the joint line and was not imprisoned in the weld line. In other words, the results indicated that the temperature gradient on the surface had a deflected angle (α) into the joint line. During the noncontact shoulder FSJ process, the tool moves forward along the joint line, and the generated heat diffuses onto the surface. The tool shoulder mainly controls the surface flow and heat generation in regular FSJ. For this reason, the surface temperature gradient in regular FSJ does not have a deflection angle [32].

As discussed before, due to the rotational direction of the tool, heat distribution on the retreating side was more significant than on the advancing side. This behavior was recorded in all samples. In the noncontact shoulder case, the generated heat inside the joint line diffused onto the surface. According to the simulation results, the deformation angle increased as the FSJ tool rotational speed increased and the tool traveling velocity decreased. The deflection angle was seemingly related to the total heat generation during the FSJ process. The deflection temperature gradient built up in the RS of the surface, and the higher amount of heat diffused in the RS was seemingly caused by the compression of hot polymer.

### 4.2. Strain Rate

**Figure 8:**
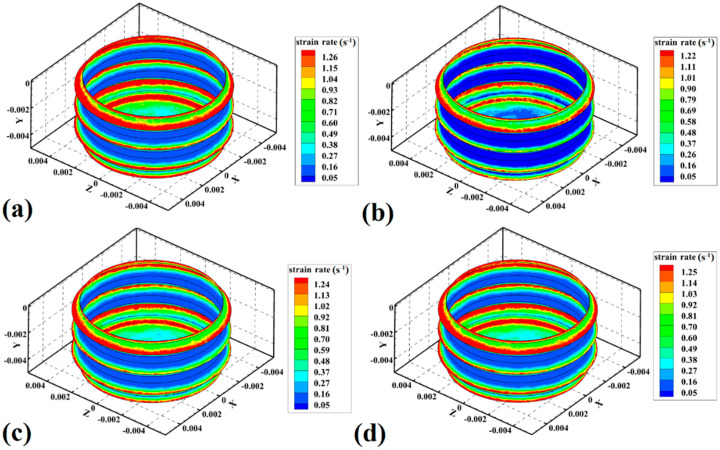
Simulation results of strain rate in (**a**) sample 1, (**b**) sample 2, (**c**) sample 3, and (**d**) sample 4.

The strain rate of materials can represent the FSJ tool’s stirring action [48]. The strain rate represents how fast the material strain occurs in the stir zone. The strain rate depends on tool rotational velocity and heat generation during the FSJ process. On the other hand, total strain represents the total amount of mixing in stir zone. The strain rate can affect the final joint properties by affecting on mixing of PA6 in the stir zone. In the noncontact shoulder FSJ process, the strain rate of PA6 in SZ was simulated, and the results are presented below. The simulation results of strain rates at SZ of samples 1, 2, 3, and 4 are depicted in Figure 8a–d, respectively. Similar to the heat generation study, the strain rate in this joint was focused on the pin of the FSJ tool. Due to the geometry of the pin, the strain rate in SZ was not uniform. The results show that the strain rate at the edges of the screw was higher than in other areas.

It was demonstrated that the tool shoulder generated the most heat during conventional FSJ of polymeric materials. This behavior was related to the greater contact area between the tool shoulder and workpiece compared to other parts of the tool. More heat generation by the shoulder caused a higher strain rate. In noncontact shoulder FSJ, the role of the shoulder in heat generation and strain rate is removed. In such a case, the pin profile determines the strain rate. For this reason, in this joint, the strain rate was concentrated around the tool pin. The central part of mixing PA6 in the stir zone was the tool’s pin. The analysis of the stir zone revealed that the strain of PA6 in the tip of the pin screw was faster than that in other areas of the pin. The results revealed that, with increasing tool rotational speed, the strain rate of PA6 in SZ increased, and, with increasing tool traveling velocity, the strain rate decreased. The results revealed that the maximum strain rate of samples 1, 2, 3, and 4 was predicted to be 1.26 s^−1^, 1.22 s^−1^, 1.24 s^−1^, and 1.25 s^−1^, respectively. The maximum strain rate was at the exterior edge of the screw. The results showed, that with increasing tool rotational velocity from 380 rpm to 500 rpm, the strain rate increased by 0.03 s^−1^. On the other hand, upon increasing the tool traveling speed from 40 mm/min to 50 mm/min, the strain rate decreased by 0.02 s^−1^.

### 4.3. Materials Velocity

**Figure 9:**
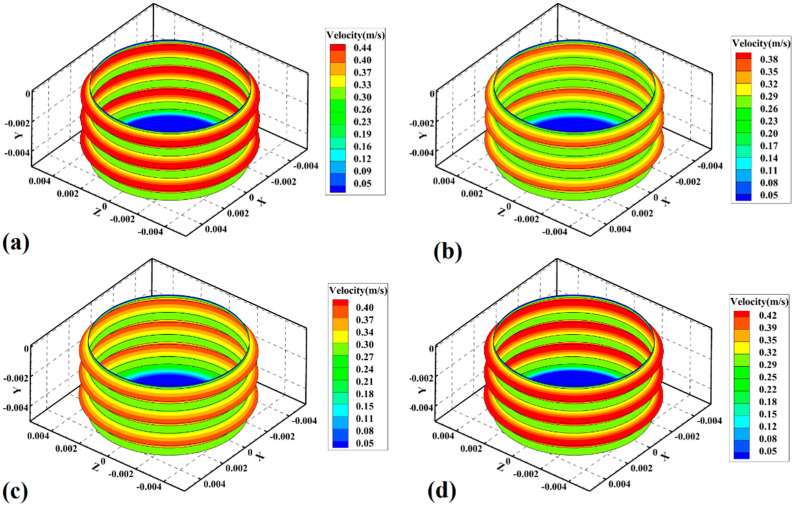
Simulation results of material velocity in (**a**) sample 1, (**b**) sample 2, (**c**) sample 3, and (**d**) sample 4.

The strain rate and heat generation contribute to the velocity of the material in SZ [49]. The velocity is related to raw material viscosity, tool shape, and process parameters. The material’s velocity is, in fact, the rotational movement of the welding material caused by the tool [50]. The simulation results of material velocity at SZ of samples 1, 2, 3, and 4 are depicted in Figure 9a–d, respectively.

Due to the lack of contact between the shoulder and PA6, the tool’s pin started to move materials on the joint line. The screw pin had the screw diameter as an exterior area and root diameter as an interior area during FSJ. As discussed before, the higher heat generation and strain rate at the exterior area (crest) of the pin screw caused the highest material velocity in the exterior area of the pin. As seen in the simulation result, the material velocity in samples 1, 2, 3, and 4 was predicted at 0.44 m/s, 0.38 m/s, 0.40 m/s, and 0.42 m/s, respectively. The maximum velocity was at the exterior edge of the screw, while the velocity of the materials at the interior area of the screw of samples 1, 2, 3, and 4 was 0.40 m/s, 0.35 m/s, 0.37 m/s, and 0.39 m/s. In this joint, the tool rotational velocity and traverse speed could determine the velocity of materials in SZ. The simulation results showed that, with an increase in tool rotational speed from 380 rpm to 500 rpm, the velocity of the materials increased by 0.04 m/s. The velocity of the material decreased by 0.02 m/s with increasing tool travel velocity from 40 mm/min to 50 mm/min.

### 4.4. Internal Material Flow

The material flow around the tool is affected by the generated heat and material velocity. The material flow path of samples can determine the shape of the stir zone and the properties of the final joint. The cross-section view of samples 1 and 2 was selected for comparison with the actual result.

**Figure 10:**
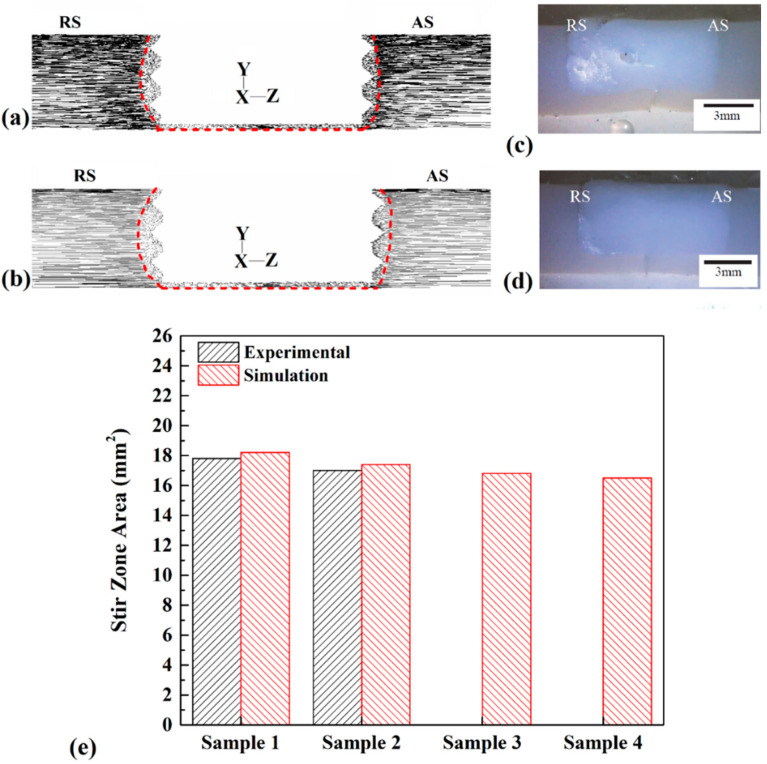
Cross-section view of materials flow path in (**a**) sample 1 and (**b**) sample 2. Cross-section view of (**c**) sample 3 and (**d**) sample 4 joints [44]. (**e**) Comparison between size of various SZ areas.

The simulation results of material flow of sample 1 and sample 2 are presented in Figure 10a,b. The results consist of flow direction around of tool. The red dashed line marks the stirring zone. The first conclusion from the results is that the stirring action of the tool increased with increasing tool rotational velocity and, consequently, more extensive SZ formed in the joint line. The results indicate that, unlike in a regular FSJ joint, the SZ in the noncontact shoulder FSJ joint had a rectangle shape. Because the shoulder did not have any contact with PA6, thermomechanical effects of the shoulder did not exist. This was the main reason for the formation of a rectangular SZ shape in this joint. Higher tool rotational speed and lower tool traveling speed increased the size of the stir zone. The similarities of the simulation results and actual samples measured and collected results are shown. The actual joints of samples 1 and 2 provided by Inaniwa et al. [44] are depicted in Figure 10c,d, respectively. The results are provided with a scale bar, allowing the area of FSJed samples to be measured. The geometrical measurement of FSJed samples is collected in Figure 10e. As expected, with increasing heat generation (at higher tool rotational velocity and lower tool traveling velocity), the size of SZ increased and vice versa.

The simulation findings revealed that the size of the SZ of samples 1, 2, 3, and 4 was 18.2 mm^2^, 17.4 mm^2^, 16.8 mm^2^, and 16.5 mm^2^. Due to simulation output, with increasing tool rotational speed, the area of the stir zone increased by 0.9 mm^2^. On the other hand, with increasing tool traveling speed, the area of the stir zone decreased by 1.4 mm^2^. The visual measurement from the actual joint revealed that the area of SZ at samples 1 and 2 was approximately 17.8 mm^2^ and 17 mm^2^, respectively. The comparison with actual results from the literature indicated good agreement with the simulation results.

## 5. Conclusions

This study successfully performed a CFD simulation of PA6 joining during noncontact shoulder FSJ. The effects of FSJ tool rotational and traverse velocity on the heat generation and material flow simulation results were validated with experimental results. The simulation output had good agreement with the experimental results. The main results of this research are presented below.

1. Due to the lack of a contact surface between the tool shoulder and workpiece, the SZ’s joint morphology and heat generation were concentrated around the pin area. The results showed that the heat generation increased with increasing tool rotational velocity. Heat generation increased by 8% when the tool velocity increased from 380 rpm to 500 rpm.
2. This joining type’s internal and surface heat flow differed from the normal FSJ process. The internal heat distribution was rectangular, and the surface temperature gradient was tilted from the joint line.
3. The velocity and strain rate of the materials in SZ were not uniform. The screw pin shape led to a higher strain rate and material velocity at the exterior area of the pin. This behavior caused the formation of a rectangular stir zone. This SZ type minimized the size of the joint line compared to the regular SZ of polymeric material welded with the conventional FSJ process.

## Figures and Tables

**Table 1:** Properties of PA6 [44].

Properties	Unit	Value
Young’s modulus	GPa	2.63
Tensile strength	MPa	67.1
Thermal conductivity	W/m·k	0.21
Glass transition temperature	°C	50
Melting temperature	°C	225
Crystallinity	%	20–25
Melt viscosity	Pa·s	2 × 10 (230 °C)

**Table 2:** Process parameters [44].

Sample	Traverse Speed (mm/min)	Rotational Speed (rpm)
1	40	440
2	50	380
3	50	440
4	50	500

## Data Availability

Not applicable.

## References

[1] Khalaf H.I., Al-Sabur R., Abdullah M.E., Kubit A., Derazkola H.A. (2022). Effects of Underwater Friction Stir Welding Heat Generation on Residual Stress of AA6068-T6 Aluminum Alloy. Materials.

[2] Aghajani Derazkola H., Simchi A., Lambiase F. (2019). Friction stir welding of polycarbonate lap joints: Relationship between processing parameters and mechanical properties. Polym. Test..

[3] Aghajani Derazkola H., Simchi A. (2020). Processing and characterizations of polycarbonate/alumina nanocomposites by additive powder fed friction stir processing. Thin Walled Struct..

[4] Iftikhar S.H., Mourad A.-H.I., Sheikh-Ahmad J., Almaskari F., Vincent S. (2021). A Comprehensive Review on Optimal Welding Conditions for Friction Stir Welding of Thermoplastic Polymers and Their Composites. Polymers.

[5] Ahmed M.M.Z., Habba M.I.A., Jouini N., Alzahrani B., Seleman M.M.E.-S., El-Nikhaily A. (2021). Bobbin Tool Friction Stir Welding of Aluminum Using Different Tool Pin Geometries: Mathematical Models for the Heat Generation. Metals.

[6] Al-Sabur R. (2021). Tensile strength prediction of aluminium alloys welded by FSW using response surface methodology—Comparative review. Mater. Today Proc..

[7] Asmare A., Al-Sabur R., Messele E. (2020). Experimental Investigation of Friction Stir Welding on 6061-T6 Aluminum Alloy using Taguchi-Based GRA. Metals.

[8] Al-Sabur R., Jassim A.K., Messele E. (2021). Real-time monitoring applied to optimize friction stir spot welding joint for AA1230 Al-alloys. Mater. Today Proc..

[9] Derazkola H.A., Khodabakhshi F., Gerlich A.P. (2020). Fabrication of a nanostructured high strength steel tube by friction-forging tubular additive manufacturing (FFTAM) technology. J. Manuf. Process..

[10] Aghajani Derazkola H., Khodabakhshi F., Gerlich A.P. (2020). Friction-forging tubular additive manufacturing (FFTAM): A new route of solid-state layer-upon-layer metal deposition. J. Mater. Res. Technol..

[11] Li T., Shi Q.Y., Li H.K., Wang W., Cai Z.P. (2008). Residual Stresses of Friction Stir Welded 2024-T4 Joints. Mater. Sci. Forum.

[12] Vahdati M., Moradi M. (2021). Statistical analysis and optimization of tensile strength of Al7075 butt joint produced by friction stir welding and submerged friction stir welding via response surface methodology and desirability approach. Amirkabir J. Mech. Eng..

[13] Pereira A.B., Fernandes F.A.O., de Morais A.B., Quintão J. (2019). Mechanical Strength of Thermoplastic Polyamide Welded by Nd:YAG Laser. Polymers.

[14] Stokes V.K. (1998). Experiments on the hot-tool welding of three dissimilar thermoplastics. Polymer.

[15] Pereira M.A.R., Amaro A.M., Reis P.N.B., Loureiro A. (2021). Effect of Friction Stir Welding Techniques and Parameters on Polymers Joint Efficiency—A Critical Review. Polymers.

[16] Maggiore S., Banea M.D., Stagnaro P., Luciano G. (2021). A Review of Structural Adhesive Joints in Hybrid Joining Processes. Polymers.

[17] Mendes N., Loureiro A., Martins C., Neto P., Pires J.N. (2014). Effect of friction stir welding parameters on morphology and strength of acrylonitrile butadiene styrene plate welds. Mater. Des..

[18] Simões F., Rodrigues D.M. (2014). Material flow and thermo-mechanical conditions during Friction Stir Welding of polymers: Literature review, experimental results and empirical analysis. Mater. Des..

[19] Azarsa E., Mostafapour A. (2014). Experimental investigation on flexural behavior of friction stir welded high density polyethylene sheets. J. Manuf. Process..

[20] Saeedy S., Givi M.K.B. (2011). Investigation of the effects of critical process parameters of friction stir welding of polyethylene. Proc. Inst. Mech. Eng. Part B J. Eng. Manuf..

[21] Panneerselvam K., Lenin K. (2012). Investigation on Effect of Tool Forces and Joint Defects During FSW of Polypropylene Plate. Procedia Eng..

[22] Panneerselvam K., Lenin K. (2014). Joining of Nylon 6 plate by friction stir welding process using threaded pin profile. Mater. Des..

[23] Lambiase F., Derazkola H.A., Simchi A. (2020). Friction Stir Welding and Friction Spot Stir Welding Processes of Polymers—State of the Art. Materials.

[24] Myalski J., Godzierz M., Olesik P. (2020). Effect of Carbon Fillers on the Wear Resistance of PA6 Thermoplastic Composites. Polymers.

[25] Khan I., Hussain G., Al-Ghamdi K.A., Umer R. (2019). Investigation of Impact Strength and Hardness of UHMW Polyethylene Composites Reinforced with Nano-Hydroxyapatite Particles Fabricated by Friction Stir Processing. Polymers.

[26] Derazkola H.A., Eyvazian A., Simchi A. (2020). Modeling and experimental validation of material flow during FSW of polycarbonate. Mater. Today Commun..

[27] Aghajani Derazkola H., Simchi A. (2018). Experimental and thermomechanical analysis of friction stir welding of poly(methyl methacrylate) sheets. Sci. Technol. Weld. Join..

[28] Sheikh-Ahmad J.Y., Deveci S., Almaskari F., Rehman R.U.R. (2022). Effect of process temperatures on material flow and weld quality in the friction stir welding of high density polyethylene. J. Mater. Res. Technol..

[29] Rehman R.U., Sheikh-Ahmad J., Deveci S. (2021). Effect of preheating on joint quality in the friction stir welding of bimodal high density polyethylene. Int. J. Adv. Manuf. Technol..

[30] Shete M.T., Yarasu R.B. (2021). Experimental investigation and finite element simulation of friction stir spot welding (FSSW) of high-density polyethylene joints. Mater. Today Proc..

[31] Khodabakhshi F., Derazkola H.A., Gerlich A.P. (2020). Monte Carlo simulation of grain refinement during friction stir processing. J. Mater. Sci..

[32] Aghajani Derazkola H., Garcia E., Elyasi M. (2021). Underwater friction stir welding of PC: Experimental study and thermo-mechanical modelling. J. Manuf. Process..

[33] Aghajani Derazkola H., Simchi A., Hamed Aghajani Derazkola A.S. (2018). Experimental and thermomechanical analysis of the effect of tool pin profile on the friction stir welding of poly(methyl methacrylate) sheets. J. Manuf. Process..

[34] Mark J.E. (1999). Polymer Data Handbook.

[35] Wypych G. (2012). Handbook of Polymers.

[36] Utracki L.A. (2002). Polymer Blends Handbook.

[37] Rosato V.D. (1997). Plastics Processing Data Handbook.

[38] Talebizadehsardari P., Musharavati F., Khan A., Sebaey T.A., Eyvaziana A., Derazkola H.A. (2021). Underwater friction stir welding of Al-Mg alloy: Thermo-mechanical modeling and validation. Mater. Today Commun..

[39] Aghajani Derazkola H., Kordani N., Aghajani Derazkola H. (2021). Effects of friction stir welding tool tilt angle on properties of Al-Mg-Si alloy T-joint. CIRP J. Manuf. Sci. Technol..

[40] Elyasi M., Derazkola H.A. (2018). Experimental and thermomechanical study on FSW of PMMA polymer T-joint. Int. J. Adv. Manuf. Technol..

[41] Aghajani Derazkola H., Khodabakhshi F. (2019). Intermetallic compounds (IMCs) formation during dissimilar friction-stir welding of AA5005 aluminum alloy to St-52 steel: Numerical modeling and experimental study. Int. J. Adv. Manuf. Technol..

[42] Memon S., Fydrych D., Fernandez A.C., Derazkola H.A., Derazkola H.A. (2021). Effects of FSW Tool Plunge Depth on Properties of an Al-Mg-Si Alloy T-Joint: Thermomechanical Modeling and Experimental Evaluation. Materials.

[43] Bokov D.O., Jawad M.A., Suksatan W., Abdullah M.E., Świerczyńska A., Fydrych D., Derazkola H.A. (2021). Effect of Pin Shape on Thermal History of Aluminum-Steel Friction Stir Welded Joint: Computational Fluid Dynamic Modeling and Validation. Materials.

[44] Inaniwa S., Kurabe Y., Miyashita Y., Hori H., Fujii H.B.T.-P. (2013). Application of Friction Stir Welding for Several Plastic Materials. Proceedings of the 1st International Joint Symposium on Joining and Welding.

[45] Iwaszko J., Kudła K. (2022). Evolution of Microstructure and Properties of Air-Cooled Friction-Stir-Processed 7075 Aluminum Alloy. Materials.

[46] Śnieżek L., Kosturek R., Wachowski M., Kania B. (2020). Microstructure and Residual Stresses of AA2519 Friction Stir Welded Joints under Different Heat Treatment Conditions. Materials.

[47] Omer M.A.E., Rashad M., Elsheikh A.H., Showaib E.A. (2022). A review on friction stir welding of thermoplastic materials: Recent advances and progress. Weld. World.

[48] Griffiths R.J., Garcia D., Song J., Vasudevan V.K., Steiner M.A., Cai W., Yu H.Z. (2021). Solid-state additive manufacturing of aluminum and copper using additive friction stir deposition: Process-microstructure linkages. Materialia.

[49] Bagheri B., Abbasi M., Hamzeloo R. (2020). The investigation into vibration effect on microstructure and mechanical characteristics of friction stir spot vibration welded aluminum: Simulation and experiment. Proc. Inst. Mech. Eng. Part C J. Mech. Eng. Sci..

[50] Memon S., Tomków J., Derazkola H.A. (2021). Thermo-Mechanical Simulation of Underwater Friction Stir Welding of Low Carbon Steel. Materials.

